# ABA Is Required for Plant Acclimation to a Combination of Salt and Heat Stress

**DOI:** 10.1371/journal.pone.0147625

**Published:** 2016-01-29

**Authors:** Nobuhiro Suzuki, Elias Bassil, Jason S. Hamilton, Madhuri A. Inupakutika, Sara Izquierdo Zandalinas, Deesha Tripathy, Yuting Luo, Erin Dion, Ginga Fukui, Ayana Kumazaki, Ruka Nakano, Rosa M. Rivero, Guido F. Verbeck, Rajeev K. Azad, Eduardo Blumwald, Ron Mittler

**Affiliations:** 1 Department of Materials and Life Sciences, Faculty of Science and Technology, Sophia University, 7–1 Kioi-cho, Chiyoda-ku, 102–8554, Tokyo, Japan; 2 Department of Plant Sciences, Mail Stop 5, University of California Davis, 1 Shields Avenue, Davis, CA, 95616, United States of America; 3 Department of Biological Sciences, College of Arts and Sciences, University of North Texas, 1155 Union Circle #305220, Denton, TX, 76203–5017, United States of America; 4 Departamento de Ciencias Agrarias y del Medio Natural, Universitat Jaume I, Campus Riu Sec, E- 12071, Castello de la Plana, Spain; 5 Centro de Edafología y Biología Aplicada del Segura, Campus Universitario de Espinardo, Espinardo, Murcia, 30100, Spain; 6 Department of Mathematics, University of North Texas, Denton, TX, 76203, United States of America; National Taiwan University, TAIWAN

## Abstract

Abiotic stresses such as drought, heat or salinity are a major cause of yield loss worldwide. Recent studies revealed that the acclimation of plants to a combination of different environmental stresses is unique and cannot be directly deduced from studying the response of plants to each of the different stresses applied individually. Here we report on the response of *Arabidopsis thaliana* to a combination of salt and heat stress using transcriptome analysis, physiological measurements and mutants deficient in abscisic acid, salicylic acid, jasmonic acid or ethylene signaling. Arabidopsis plants were found to be more susceptible to a combination of salt and heat stress compared to each of the different stresses applied individually. The stress combination resulted in a higher ratio of Na^+^/K^+^ in leaves and caused the enhanced expression of 699 transcripts unique to the stress combination. Interestingly, many of the transcripts that specifically accumulated in plants in response to the salt and heat stress combination were associated with the plant hormone abscisic acid. In accordance with this finding, mutants deficient in abscisic acid metabolism and signaling were found to be more susceptible to a combination of salt and heat stress than wild type plants. Our study highlights the important role abscisic acid plays in the acclimation of plants to a combination of two different abiotic stresses.

## Introduction

The evolution of land plants was accompanied by the acquisition of acclimation and adaptation mechanisms to fluctuating environmental conditions. In the last decade, multiple pathways underlying the response of plants to abiotic stresses such as drought, salinity and heat were uncovered. The majority of these were studied in plants subjected to a single abiotic stress applied under controlled condition. In nature, however, different stresses can occur simultaneously impacting plants in a manner that differs from that caused by a single stress condition applied individually [1–5]. The transcriptome of plants grown under a combination of drought and heat stress was shown for example to be different from that of plants subjected to drought or heat stress applied independently [2, 6, 7]. A recent transcriptome analysis of *Arabidopsis thaliana* plants subjected to several different abiotic and biotic stresses as single stresses, or in combination, revealed that approximately 60% of the transcripts expressed under stress combinations cannot be deduced from studying the single stresses comprising the stress combination individually [8]. In addition, specific physiological and molecular responses of plants to different combinations of abiotic and biotic stresses were identified [9–14]. These findings indicated that some of the mechanisms required for the acclimation of plants to a combination of different stresses are distinct from those required for acclimation to a single stress condition [2, 4, 5, 15].

Salinity and heat stress are a major cause of damage to agricultural crops worldwide [15, 16]. Salinity stress can cause Na^+^ toxicity that affects K^+^ uptake, and results in the impairment of enzymatic activities as well as inhibition of metabolic pathways [17, 18]. Heat stress can cause alterations in membrane fluidity that affect the function of membrane-bound ion transporters [19–21]. Some of the plant responses to salinity and heat stress are regulated by abscisic acid (ABA). Abscisic acid mediates stomatal closure to prevent water loss caused by osmotic stress under high salt stress [22]. In contrast to salinity stress, ABA-dependent stomatal closure might be disadvantageous for the acclimation of plants to heat stress because it could prevent leaf cooling via transpiration. In addition to its role in stomatal responses, ABA was shown to play an important role in the regulation of transcript expression during heat stress [23]. A recent study demonstrated that hydrogen peroxide can enhance ABA-dependent expression of heat shock protein 70 (HSP70) and enhance the tolerance of plants to heat stress [23]. In addition, temporal and spatial interactions of ABA with reactive oxygen species (ROS) signals (the ROS wave) were shown to play a key role in the regulation of systemic acquired acclimation of plants to heat stress [24, 25].

It was previously thought that the harmful effects of salinity stress could be accelerated when this stress is combined with heat stress because enhanced transpiration could increase uptake of salt into the upper parts of the plant [5, 26, 27]. A recent study demonstrated nonetheless that a combination of heat and salt stress had less harmful effects compared to salinity alone in tomato [28]. It was proposed that accumulation of glycine betain and trehalose could be a key process in the response of tomato to a combination of heat and salinity [28]. The accumulation of these compounds under the stress combination was shown to be correlated with the maintenance of a lower Na^+^/K^+^ ratio, higher cellular water content in cells and improved photosynthetic performance compared to plants subjected to salinity stress alone [28].

Here we used transcriptomics, physiology and genetic analyses to study the response of *Arabidopsis thaliana* to a combination of salinity and heat stress. Our study revealed that in contrast to tomato [28], Arabidopsis plants (ecotypes Colombia or Landsberg erecta) were more susceptible to the combination of salt and heat stress than to each of the different stresses applied individually. Interestingly, the stress combination caused a decrease in the level of K^+^ ions in leaves resulting in a higher ratio of Na^+^/K^+^ without altering the level of Na^+^ ions compared to that observed in salt stress alone. Our RNA-Seq analysis revealed that the expression of many transcripts in Arabidopsis was specifically altered in response to the stress combination (699 transcripts were significantly up regulated and 585 were significantly downregulated), and that the expression of many of these transcripts was associated with the plant hormone ABA. In support of this finding mutants deficient in ABA metabolism (*aba1*) and signaling (*abi1*) were found to be more susceptible to the stress combination than wild type plants. Our study highlights the important role of ABA in the acclimation of plants to abiotic stress combinations.

## Materials and Methods

### Plant material and growth conditions

*Arabidopsis thaliana* (cv Columbia) Col, *sid2* [29], *lox3* [30], *ain1-1* [31], *mbf1c* [32], *apx1* [24, 33], Ler (cv Landsberg erecta), *aba1* and *abi1* [34] were grown on soil mixture (MetroMix 200, SUN GRO) in 240-cm^2^ inserts under controlled conditions: 21°C, 12-h light cycle, 100 μmol m^-2^s^-1^, and relative humidity of 70% (E-30 AR-66, Percival Scientific) as described before [7].

### Stress treatments

Two different stress treatments were used in this study: A 3 day treatment that was used to study survival, growth, and chlorophyll content following stress combination, and a 1 h treatment that was used to conduct RNA-Seq, qRT-PCR, and Na^+^ and K^+^ analysis following stress combination. All treatments were performed in parallel. Salinity stress was imposed on 12-d-old plants by adding 150mM NaCl to the nutrient solution [35] for 15–17 days. Heat stress was applied by transferring 25-d-old plants grown in the presence or absence of salt stress to a growth chamber with the following cycle; 06:00–09:00, 21°C; 09:00–17:00, 43°C; 17:00–09:00, 21°C. Plants were grown for a total of 3 days under these temperature conditions. The 12h light period was imposed from 08:00 to 20:00. As shown in Figure A in S1 Fig, this treatment resulted in a daily 1 h ramping of temperature from 20 to 43°C that was followed by a 7 h treatment at 43°C and a 1 h decline of temperature from 43 to 20°C. Following the 3 day stress cycle plants were recovered under controlled conditions for 7 days and survival rate, growth parameters, and chlorophyll concentration were scored. Because we were not able to observe differences in growth parameters between control, heat, salinity or the stress combination immediately after the stress treatment, these parameters were scored following the 7-day recovery. For plant survival measurements, plants were scored as survived if their meristem and the 3 newest leaves were green after the 7 day recovery period.

For RNA-Seq, qRT-PCR, and Na^+^ and K^+^ analyses, 25-d-old plants grown in the presence or absence of salinity stress as described above were transferred to a growth chamber set for 44°C, incubated for 1h and sampled. All plants, i.e. salt-stressed plants, plants subjected to heat stress without salt, salt- and heat-stressed plants, and control plants kept at 21°C were sampled at the same time for analysis. As shown in Figure B S1 Fig, this treatment resulted in a 30 min ramping of temperature from 20 to 43°C that was followed by a 30 min treatment at 42.5°C. A portable USB datalogger (Model OM-EL-USB-2-LCD-PLUS, OMEGA Engineering, INC., Stamford, Connecticut, USA) was used to measure growth chamber internal temperature and humidity parameters.

### Molecular and physiological analyses

Total chlorophyll was determined according to [36]. The expression of several transcripts was examined by quantitative real-time PCR [37] using the StepOnePlus real-time PCR system (Applied Biosystems). The quantitative PCR data were analyzed with StepOnePlus software v2.0.1 (Applied Biosystems). Threshold cycle values for genes encoding ABA response protein, Glyoxylase 17, RbohD, NCED3, CAT2 and Cor78 were calculated with the cycle threshold of EF1-a as an internal control. Primer pairs used for amplifications are shown in S16 Table online.

### LA-ICP-MS analysis

For elemental analysis of Na and K, leaves were divided into five sections from the tip to the base, where the leaf and stem meet. Laser ablation-inductively coupled plasma-mass spectrometry analysis (LA-ICP-MS) was performed using an in-house Peltier cooled ablation cell, as described before [38], constructed for use with an UP-213 laser system (New Wave Research, Fremont, CA) coupled to a Bruker, (formerly Varian 820MS) quadrupole ICP-MS to analyze 23Na and 39K ions within the Arabidopsis leaves. A whole, frozen, Arabidopsis leaf was placed on a square glass cover slip (No.1, 22mm x 22mm, Corning, Corning, NY). The cover slip was placed directly atop the Peltier cooling device of the ablation cell, inserted into the laser ablation chamber, and the cell was then purged with He gas. The leaf was then located and brought into focus using the motorized stage and visualized with a CCD camera. Once in focus, two straight-line continuous raster patterns were created for each of the five leaf sections, one to the left of center and one to the right of center, for a total of ten ablations per leaf. The laser settings for each raster consisted of a 10s laser warm-up followed by laser ablation with the following settings: laser spot size of 100 μm, a 150 μm raster spacing, scan rate of 100 μm/s, 10Hz repetition rate, and a laser output of 30%.

### RNA-Seq

For RNA-Seq analysis, three independent biological replicates, each composed of leaves pooled from at least 20 different plants grown as described in “Stress treatment”, were used per experimental condition. Total RNA was isolated and purified as described previously [24] and RNA-Seq analysis was conducted using an Illumina HiSeq2000 at the University of Wisconsin-Madison Biotechnology Gene Expression Center (http://www.biotech.wisc.edu/services/gec). GO annotations of the transcripts identified by our RNA-seq analyses were obtained from TAIR (https://www.arabidopsis.org/tools/bulk/go/index.jsp). RNA-Seq data was deposited in NCBI GEO repository under the accession/reference number GSE72806.

### Bioinformatics analysis

GO annotations of the transcripts identified by RNA-Seq analyses were obtained from The Bio-Analytic Resource for Plant Biology (http://bar.utoronto.ca/). The overlap between transcripts up-regulated in leaves in response to short-term high light exposure and transcripts up-regulated in response to ABA, ethylene (ACC), brassinolide (BL), cytokinin (CK), gibberellin (GA), auxin (IAA), MJ, SA, H_2_O_2_, O_2_^-^ or ^1^O_2_ [39–43], or in response to different abiotic stresses [7, 8, 44–52] was determined as previously described [33, 37].

### Statistical analysis

We performed next generation RNA sequencing (RNA-Seq) for differential expression profiling and characterization of transcript processing events. Three biological replicates were obtained as described above. Single-end Illumina sequencing generated on average 14 million reads per sample, with each sequence read of length 50 nucleotides. We utilized the services of frequently used, publicly available RNA-Seq analysis software, namely, Bowtie [53], Tophat [54] and Cufflinks [55], for alignment of single-end reads onto the reference genome, parsing the alignment to infer the exon-exon splice junctions, and performing the differential expression analysis of annotated genes. Pre-alignment filtering of the Illumina data was performed with Tophat and Bowtie programs that perform pre-alignment filtering. Only clean reads were kept for further downstream analysis. TAIR9 genome-build, Bowtie version 0.12.8.0, Samtools version 0.1.18.0; TopHat run version 2.0.4 and Cufflinks version 2.0.2 were used using default parameter settings (program-author-provided). Transcripts expressing differentially in two (or more) conditions were identified by examining the difference in their abundance under the two conditions. The abundance of a transcript was measured in terms of “Fragments Per Kilobase of transcript per Million fragments mapped” (FPKM), normalized for the transcript length and total number of cDNA fragments for a sample replicate. The difference in expression was obtained as the log of fold change in abundance between the two conditions. Statistical significance test for differential expression of each transcript was performed based on a negative binomial model estimated from the data [55]. The fold change of genes with multiple isoforms was assessed by summing up the FPKMs for all isoforms of a gene and then measuring the difference between the two conditions [55]. Although this type of analysis excludes the effects of differential splicing, it provides a measure for differential expression. Other statistical analyses were performed by one-tailed Student’s t-test as previously described [56]. Results are presented as the Mean ± SD or SE (* P<0.05; ** P<0.01).

## Results

### Growth and survival of Arabidopsis plants subjected to a combination of salinity and heat stresses

**Fig 1:**
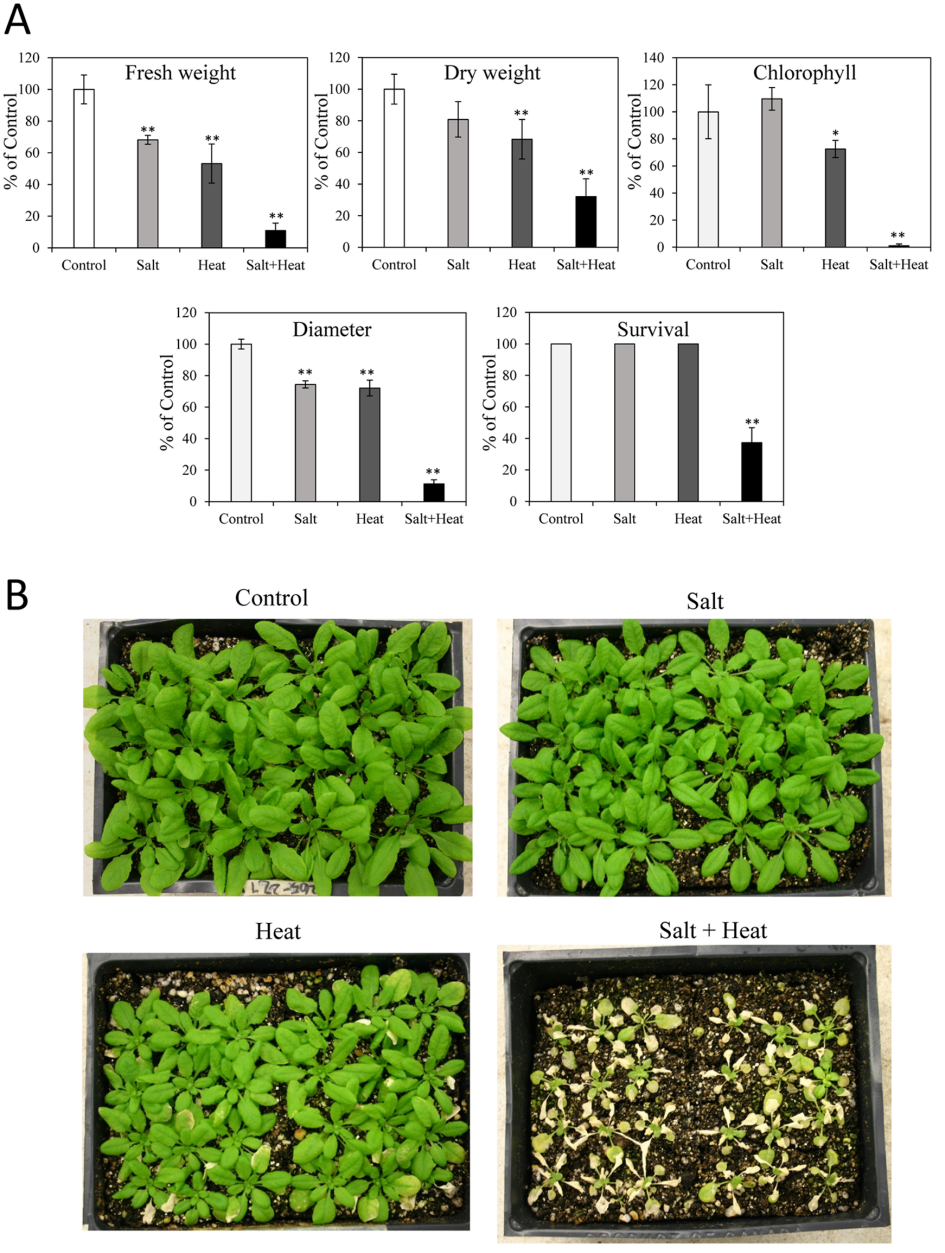
Acclimation of *Arabidopsis thaliana* ecotype Col plants to salt, heat and a combination of salt and heat stress. (A) Growth parameters, chlorophyll content and survival of plants subjected to salt, heat and a combination of salt and heat stress. (B) Representative images of plants subjected to the different stresses. * or **, Student’s t test significant at *P < 0.05 or **P < 0.01 compared to control (n = 30). Error bars represent SD.

**Fig 2:**
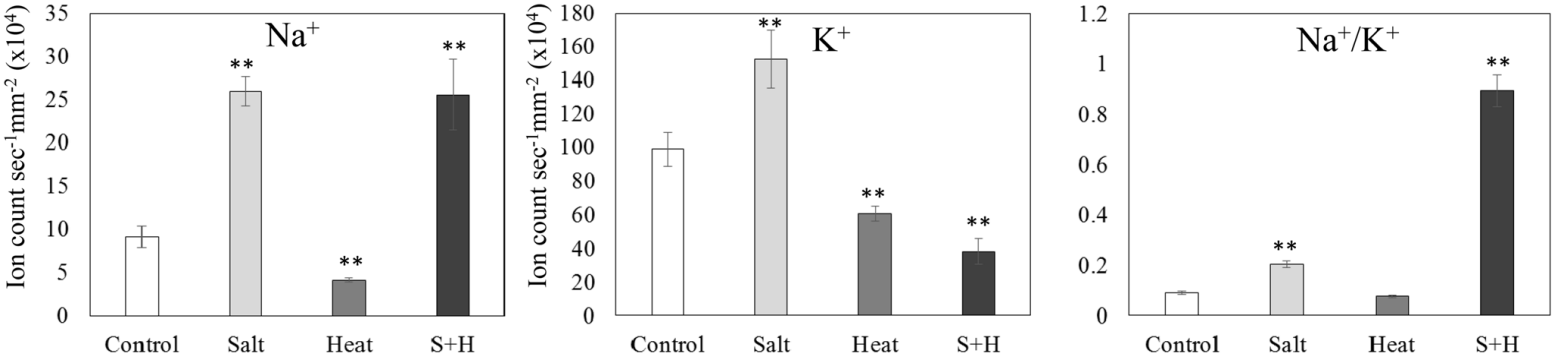
Na^+^ and K^+^ ion content, and Na^+^/K^+^ ratio in leaves of Arabidopsis plants subjected to salt, heat and a combination of salt and heat stress. **, Student’s t test significant at *P<0.01 compared to control (n = 27). Error bars represent SE.

We measured growth and physiological parameters of Arabidopsis (Col) subjected to salinity, heat stress and a combination of salinity and heat stress (Figs 1 and 2). The three stress treatments resulted in a significant decrease in both shoot fresh and dry weight as well as rosette diameter, with the largest decrease caused by the stress combination (Fig 1). Although 100% of plants were able to survive salinity or heat stress applied individually, only about 40% of plants were found to survive the stress combination. In addition, leaf chlorophyll content was significantly decreased under both heat stress and a combination of salinity and heat stress, but not under salinity alone, with the largest decrease observed under the heat and salinity stress combination (Fig 1).

### Na^+^ and K^+^ contents in plants grown under salinity, heat and a combination of salinity and heat

Na^+^ and K^+^ concentrations were measured in leaves of Arabidopsis plants subjected to salinity, heat and a combination of salinity and heat stresses (Fig 2). As expected, compared with plants grown under controlled conditions, Na^+^ concentration was approximately 200% higher in plants subjected to salinity. The K^+^ concentration in salt-treated plants was also elevated, but only by 60% compared to control. In heat-treated plants, both the Na^+^ and K^+^ contents were reduced slightly compared to control. In plants grown under a combination of salinity and heat stress, the Na^+^ concentration increased to almost the same level as in plants subjected to salinity alone, however the K^+^ concentration was significantly reduced compared to the K^+^ content of salt-treated plants. These changes in Na^+^ and K^+^ contents resulted in a pronounced increase in the Na^+^/K^+^ ratio of plants grown under a combination of heat and salinity stress.

### Transcriptomic analysis of plants subjected to salinity, heat and a combination of salinity and heat stress

**Fig 3:**
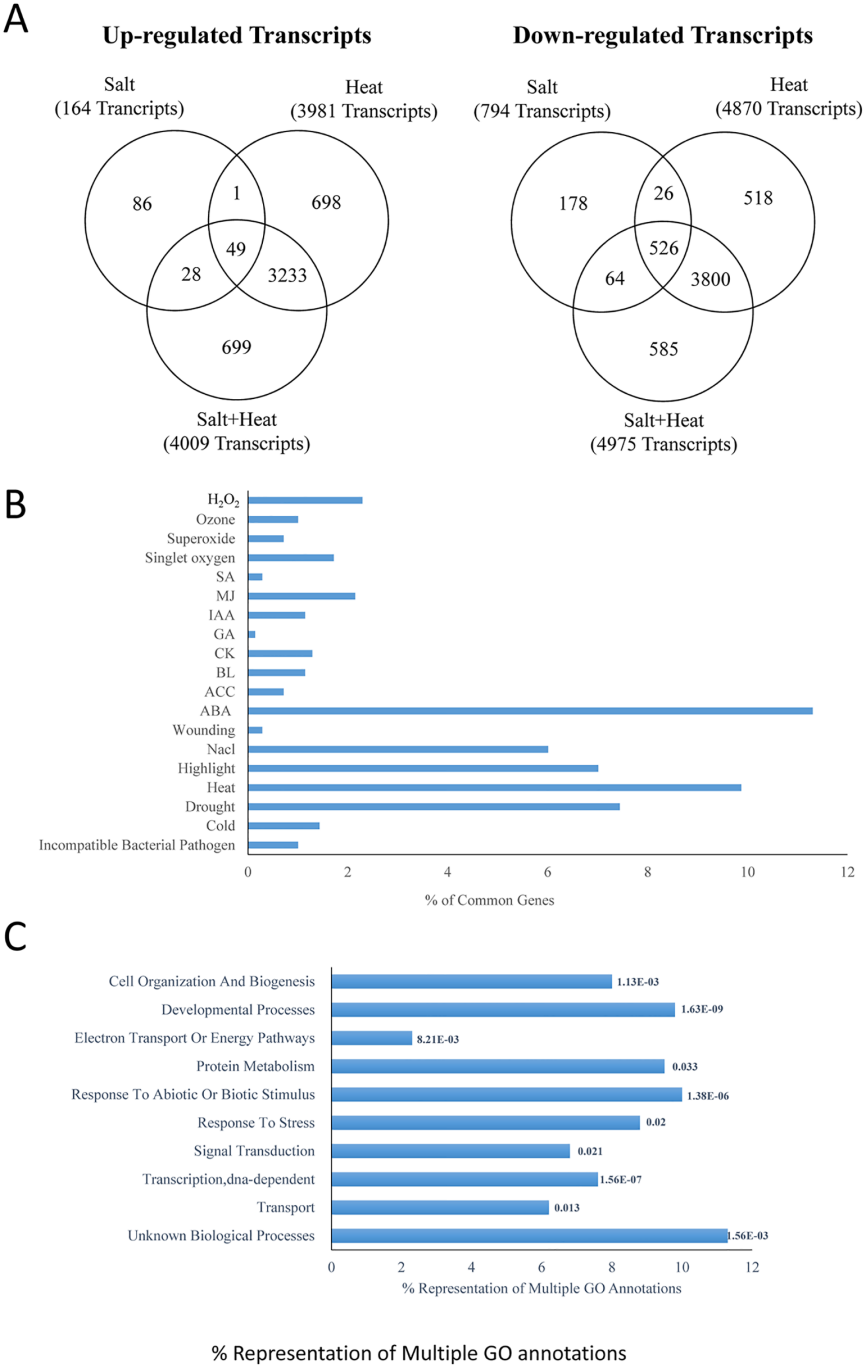
Transcriptomic analysis of Arabidopsis plants subjected to salt, heat and a combination of salt and heat stress. (A) Venn diagram showing the overlap between transcripts significantly up- or down-regulated in response to salt, heat and a combination of salt and heat stress. (B) Representation of ROS-, hormone-, and abiotic/biotic stress- response transcripts within the group of transcripts that are significantly up-regulated in response to the combination of salt and heat stress. (C) GO annotation of the transcripts specifically up-regulated in response to a combination of salt and heat stress.

To examine global changes in the transcriptome of Arabidopsis leaves subjected to salinity, heat stress and their combination, we performed RNA-Seq analysis (Fig 3A and S1–S14 Tables). As shown in Fig 3A, 50 transcripts were common between the 164 transcripts significantly up-regulated by salinity and the 3981 transcripts up-regulated by heat stress. An overlap of 552 transcripts was observed between the 794 and 4870 transcripts that were significantly down-regulated by salinity or heat stress, respectively. Compared to non-stressed plants, the steady-state level of 4009 transcripts was up-regulated and 4975 transcripts were down-regulated by the combination of salinity and heat stress. Out of the 4009 up-regulated transcripts, 77 were also up-regulated by salinity. In contrast, 3282 of the 4009 up-regulated transcripts were common to both the stress combination and the heat stress treatment. A similar high number of down-regulated transcripts (4326) were common between the heat stress and the stress combination. In addition to shared transcripts differentially expressed during salinity or heat stress, the combination of salinity and heat stress contained 699 transcripts specifically up-regulated, and a further 585 transcripts specifically down-regulated, in response to the stress combination (Fig 3A). The transcriptome of plants subjected to a combination of salinity and heat stress was therefore significantly different from that of plants subjected to heat or salt stress, harboring many transcripts that specifically responded to the stress combination.

To corroborate the transcriptomics results obtained by RNA-Seq analysis, we quantified the expression of several transcripts using qPCR (S2 Fig). Two transcripts, encoding an ABA response protein (At3g02480) and Glyoxylase 17 (At1g80160) were upregulated by heat stress and a combination of salinity and heat stress; Two transcripts, NCED3 (At3g14440) and Cor78 (AT5G52310) were upregulated by all stresses employed in this study; and RbohD (At5g47910) was specifically upregulated by a combination of salinity and heat stress. In contrast, the expression of CAT2 (At4g35090) was not altered during salinity but decreased in plants subjected to heat or a combination of salinity and heat stress.

To dissect the transcriptome response of Arabidopsis to a combination of salt and heat stress we identified and categorized into groups the 699 transcripts that were specifically up-regulated in response to a combination of salt and heat stress. As shown in Fig 3B, compared to all other hormone-response transcripts, those associated with ABA responses were the most highly represented (over 11% of all transcripts) in this group. In contrast to ABA response transcripts, GA and SA response transcripts that could belong to pathways that antagonize ABA function [57, 58] were the least represented compared to other hormone-response transcripts (Fig 3B and S3 Fig). Surprisingly, many of the transcripts that were specifically up regulated in response to a combination of salt and heat were also up regulated in response to light stress. Out of the 699 transcripts that were specifically up-regulated by the combination of salinity and heat stress, 104 transcripts were identified by other studies as salt or heat response transcripts. These were therefore removed from the list of salt and heat combination specific transcripts. The resulting 595 transcripts (S15 Table) were then subjected to a GO annotation analysis. As shown in Fig 3C, transcripts of unknown function and transcripts involved in transcription or response to abiotic or biotic stimulus were significantly more represented in this data set.

### ABA is required for acclimation of plants to a combination of salinity and heat stress

**Fig 4:**
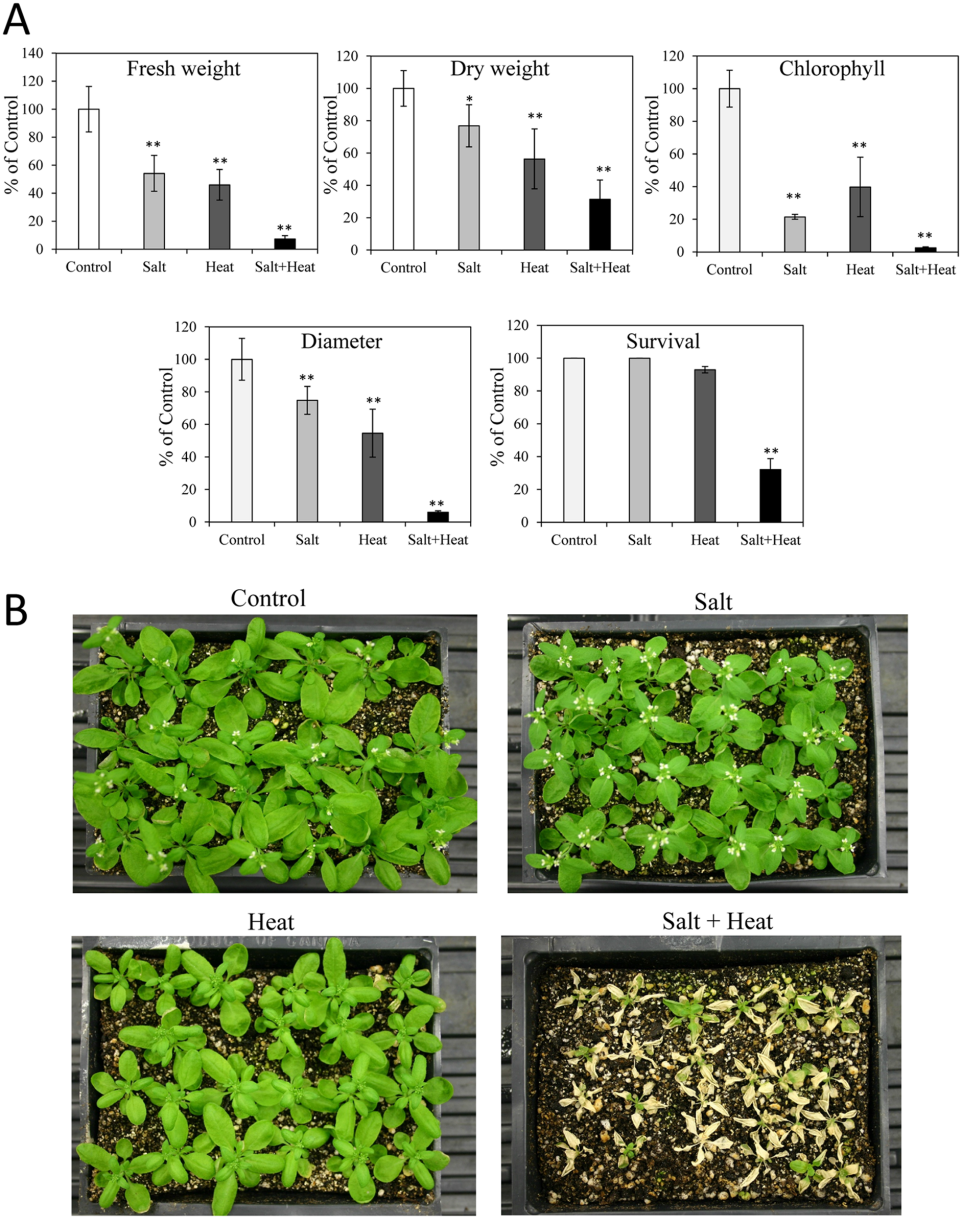
Response of Arabidopsis ecotype Ler plants to salt, heat and a combination of salt and heat stress. (A) Growth parameters, chlorophyll content and survival of plants subjected to salt, heat and a combination of salt and heat stress. (B) Representative images of plants subjected to the different stresses. * or **, Student’s t test significant at *P < 0.05 or **P < 0.01 compared to control (n = 30). Error bars represent SD.

**Fig 5:**
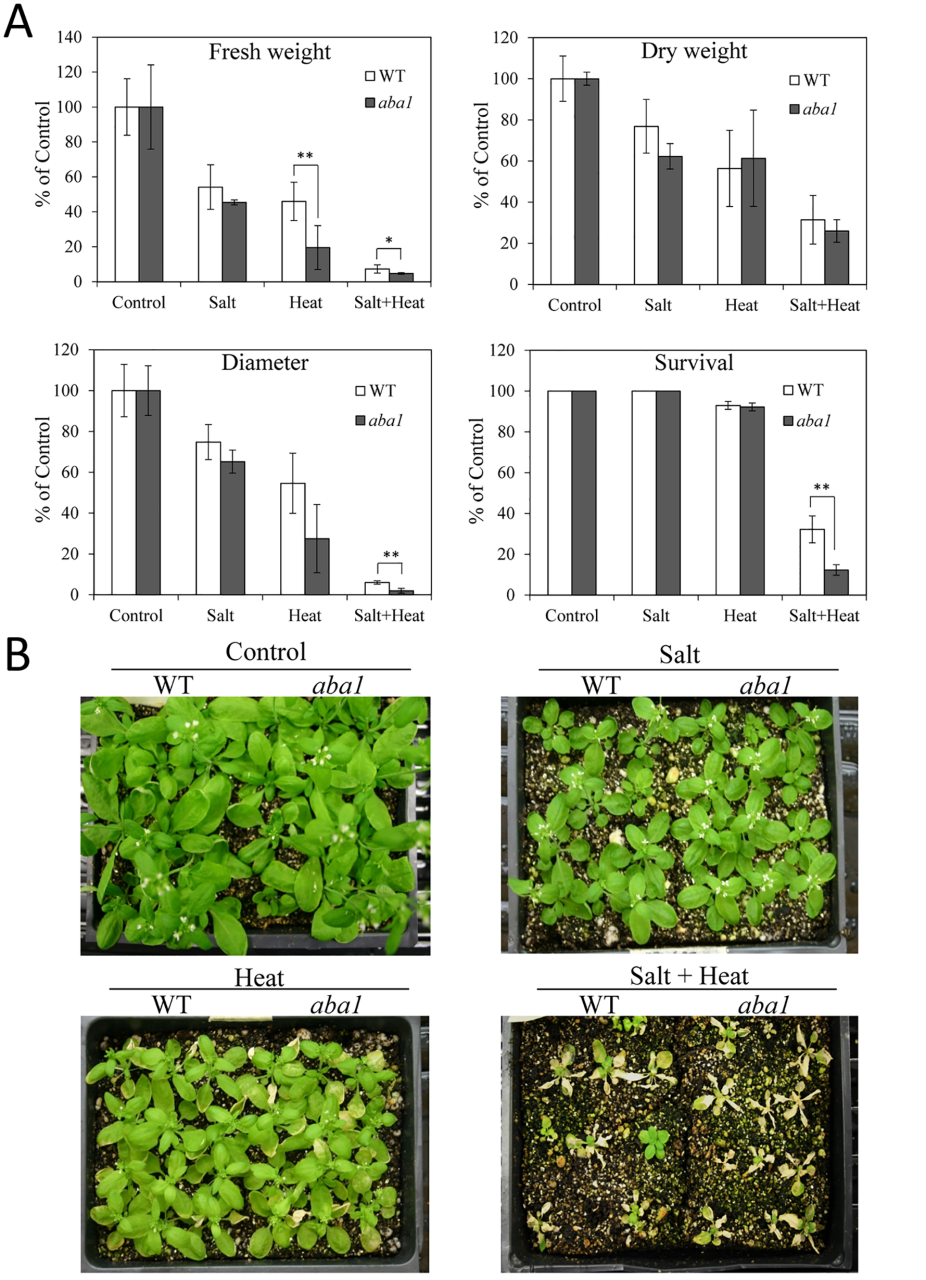
Response of *aba1* plants to salt, heat and a combination of salt and heat stress. (A) Growth parameters and survival of plants subjected to salt, heat and a combination of salt and heat stress. (B) Representative images of plants subjected to the different stresses. * or **, Student’s t test significant at *P < 0.05 or **P < 0.01 compared to WT (n = 30). Error bars represent SD. Absolute values for the graphs shown in (A) are presented in S9 Fig.

**Fig 6:**
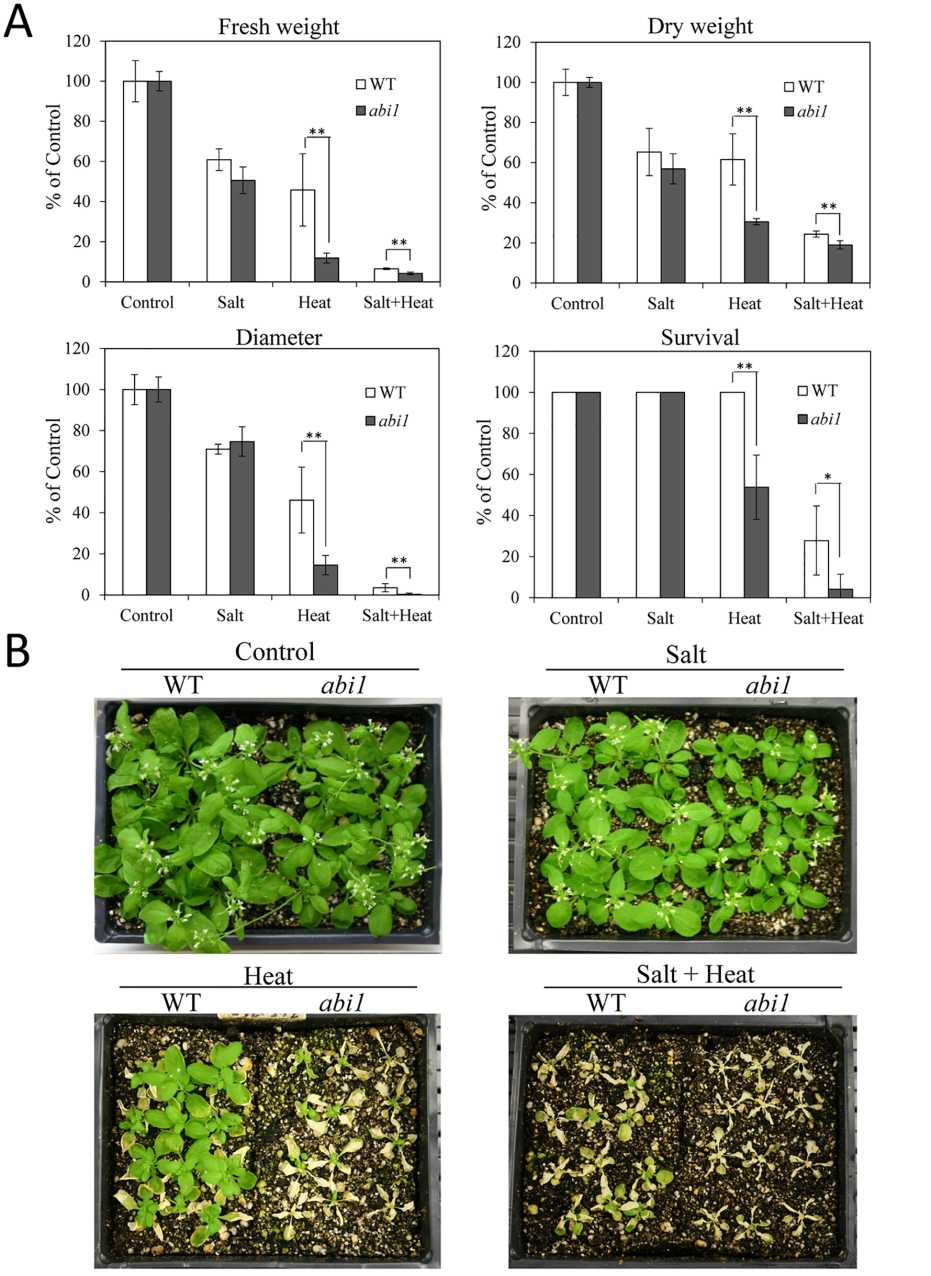
Response of *abi1* plants to salt, heat and a combination of salt and heat stress. (A) Growth parameters and survival of plants subjected to salt, heat and a combination of salt and heat stress. (B) Representative images of plants subjected to the different stresses. * or **, Student’s t test significant at *P < 0.05 or **P < 0.01 compared to WT (n = 30). Error bars represent SD. Absolute values for the graphs shown in (A) are presented in S10 Fig.

The high representation of ABA-response transcripts, compared to all other hormone-response transcripts, among the salt and heat combination specific transcripts (Fig 3B) prompted us to study the response of different mutants impaired in hormone signaling/metabolism to a combination of salt and heat. We therefore compared the response of wild type plants to that of mutants impaired in ABA, ethylene, salicylic acid (SA), or jasmonic acid (JA) signaling to salinity, heat stress and their combination (Figs 4–6 and S4–S6 Figs).

Because the genetic background of the mutants impaired in ABA metabolism/signaling is Landsberg erecta (Ler), we first studied the response of Ler to salinity, heat stress and their combination (Fig 4). Similar to Col plants, either salinity or heat stress alone and the combination of salinity and heat stress all significantly decreased Ler fresh and dry weight and rosette diameter, with the more severe effects observed in response to the stress combination. The reduced survival of Ler in response to a combination of salinity and heat stress was almost the same as that of Col plants (40%; Figs 1 and 4). Ler plants were however slightly more sensitive to heat stress compared to Col with approximately 3–4% of Ler plants not surviving the heat stress (Figs 1 and 4). In addition, the chlorophyll content of Ler decreased significantly under the three stress treatments (Fig 4).

To study the involvement of ABA in the response of plants to salinity, heat stress and their combination, we studied the acclimation of the *aba1* mutant that is deficient in ABA biosynthesis, to the single and combined stresses (Fig 5). Compared to wild type Ler plants, *aba1* plants showed reduced fresh weight, plant diameter and survival rate in response to a combination of salinity and heat stress. Fresh weight and diameter of *aba1* plants was also reduced under heat stress, but the difference in diameter between wild type and *aba1* was not statistically significant. To further investigate the involvement of ABA in the response of plants to salinity, heat stress and their combination, we studied the acclimation of the *abi1* mutant, deficient in ABA signaling, to the same stresses (Fig 6). Compared to wild type Ler plants, *abi1* plants displayed reduced fresh weight, plant diameter and survival rate in response to a combination of salinity and heat stress. In contrast to *aba1*, the dry weight of *abi1* plants was however significantly reduced compared to wild type under both heat and heat and salinity combination.

In contrast to mutants deficient in ABA synthesis or signaling (Figs 5 and 6), mutants deficient in the synthesis of, or response to, SA, JA, or ethylene were found not to have a significant difference compared to wild type (cv Columbia) in their acclimation to salinity, heat stress or their combination (S4–S6 Figs).

### The acclimation of Arabidopsis to salt and heat stress combination is different from that to drought and heat stress combination

To compare between the acclimation of Arabidopsis plants to salt and heat stress combination and the acclimation of Arabidopsis plants to drought and heat combination, we studied the acclimation of plants altered in the expression of two different proteins important for Arabidopsis tolerance to a combination of drought and heat, to a combination of salinity and heat stresses (S7 and S8 Figs). Plants that constitutively overexpressed the multiprotein bridging factor 1c (MBF1c) were previously found to be more tolerant to a combination of osmotic and heat stress [32]. In contrast, Arabidopsis plants deficient in cytosolic ascorbate peroxidase 1 (APX1) were more sensitive to a combination of drought and heat stress [59]. We therefore tested the acclimation of plants deficient in MBF1c (*mbf1c*) or APX1 (*apx1*) to the combination of salt and heat stress. Both mutants were similar to wild type in their acclimation to salt, heat and the combination of salinity and heat stress (S7 and S8 Figs), suggesting that the acclimation of Arabidopsis plants to a combination of salt and heat stress is mediated by mechanisms that are different from those involved in the acclimation of Arabidopsis to a combination of drought and heat stress.

**Fig 7:**
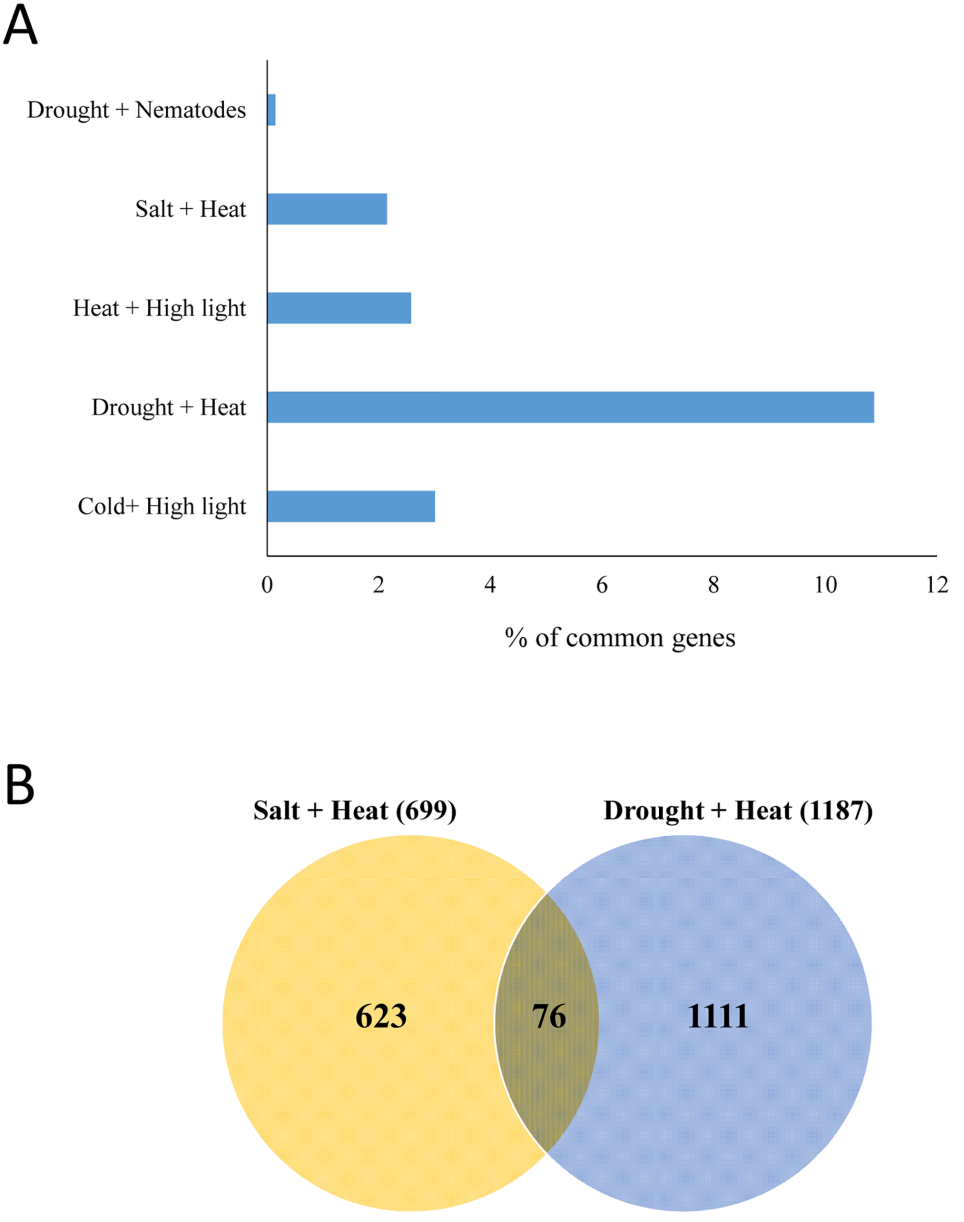
Specificity of stress combination-specific transcripts. (A) Overlap between transcripts specifically and significantly up-regulated in response to a combination of salt and heat stress, and transcripts specifically and significantly up-regulated in response to other stress combinations. (B) Venn diagram showing the overlap between transcripts specifically and significantly up-regulated in response to a combination of salt and heat stress, and transcripts specifically and significantly up-regulated in response to a combination of drought and heat stress. References used for the meta-analysis are [7, 8, 52].

To further compare between the acclimation of Arabidopsis to salt and heat stress combination and the acclimation of Arabidopsis to drought and heat combination, as well as other stress combinations, we compared the set of transcripts specifically up-regulated in response to the salt and heat stress combination (Fig 3A), to that of transcripts specifically up-regulated in Arabidopsis in response to other stress combinations [7, 8, 52]. As shown in Fig 7, little overlap was found between the different sets of transcripts specifically up-regulated in response to salt and heat (this study), heat and high light, salt and high light, drought and heat, cold and high light, or drought and nematodes (Fig 7A). As shown in Fig 7B, only 76 transcripts were common between the response of Arabidopsis to salt and heat combination and the response of Arabidopsis to drought and heat combination, supporting our findings with the *mbf1c* or *apx1* mutants (S7 and S8 Figs).

## Discussion

The ability to sense and acclimate to a combination of different abiotic stress conditions is particularly important for field-grown plants. In the case of salinity and heat stress combination the increased transpiration associated with heat stress is thought to worsen the harmful effects of salinity because it could increase the uptake of salt into leaves and roots under the stress combination [26, 27]. In our study, the growth and chlorophyll content of plants was indeed significantly more impacted by the combination of heat and salt stresses, compared to each of these conditions applied individually, supporting a negative interaction between these two stresses (Figs 1 and 4). Interestingly, the stress combination was not found to elevate the level of Na^+^ in leaves compared to that found in salt treated plants in the absence of heat, but instead resulted in an altered K^+^ levels (Fig 2). In contrast to the harmful effects of the salinity and heat stress combination on Arabidopsis plants reported in this study (Figs 1 and 4), a similar stress combination was previously reported to have less damaging effects on tomato plants compared to salt stress applied individually [28]. The difference in tolerance to the salinity and heat combination between tomato and Arabidopsis could suggest that different plants respond differently to the same stress combination. In addition, differences in the timing and intensity of the salinity and heat stress treatments could explain the differences between the different experimental systems. One apparent difference between the response of Arabidopsis and tomato plants might be their Na^+^ and/or K^+^ homeostasis. When the different plants were grown under a combination of salinity and heat stress, tomato displayed a reduction in the Na^+^/K^+^ ratio [28], whereas in Arabidopsis the Na^+^/K^+^ increased (Fig 2). In tomato, the inhibition of Na^+^ transport and uptake was shown to be a key mechanism of protection against a combination of salinity and heat stress [28]. In contrast, in Arabidopsis, Na^+^ uptake under salinity was not affected by the heat stress combination, whereas K^+^ uptake increased under salt stress but was markedly reduced by the combination of salinity and heat stress. The effects of the stress combination on Arabidopsis might therefore be associated with mechanisms altering K^+^ and not Na^+^ levels. Given the importance of K^+^ homeostasis under salinity [60], the mechanisms controlling Na^+^ and K^+^ homeostasis of different plants under a combination of salinity and heat stress should be addressed in future studies.

A considerable number of transcripts were specifically up-regulated in response to a combination of salt and heat stress, suggesting that the response of plants to this stress combination includes unique pathways that are not directly involved in the acclimation of plants to salt or heat stress applied individually (Fig 3). An extensive overlap of differentially expressed transcripts in response to salinity and heat stress combination and heat stress was nonetheless found (Fig 3A). This finding could indicate that heat stress responses might dominate the acclimation response of Arabidopsis to the salinity and heat stress combination. At least when it comes to the role of ABA in these responses, our results support such an interaction because ABA was found to be involved in the acclimation of plants to heat stress, as well as a combination of salinity and heat stress (Figs 5 and 6). Involvement of heat response pathways in the acclimation of plants to salt stress was previously suggested by the up-regulation of HSFs and HSPs in response to both salinity and heat stress [61]. In addition, overexpression of Arabidopsis HSP17.8 in lettuce resulted in enhanced tolerance to salt stress [62].

The involvement of ABA in the acclimation of plants to abiotic stress combination, demonstrated with two different mutants impaired in ABA metabolism/signaling (Figs 5 and 6), is a new finding described in this manuscript. This involvement could be related to the function of ABA in the regulation of gene expression during stress combination, or reflect the role ABA plays in stomatal regulation during stress. Further studies are required to address these questions. In addition, further studies are required to examine what other stress combinations could require ABA signaling in different plants. In light of previous studies that suggested an antagonistic relationship between ABA and GA or SA signaling [57, 58], the higher proportion of ABA response transcripts relative to GA and SA response transcripts in the data sets of transcripts specifically upregulated in response to a combination of salt and heat stress (Fig 3 and S3 Fig) further supports the involvement of ABA in the response of plants to this stress combination.

Previous studies uncovered several signaling pathways involved in the response of plants to a combination of drought and heat stress. These included the function of MBF1c, ethylene and APX1 [7, 59, 63]. Results presented in this manuscript do not however support a role of these mechanisms in the acclimation of plants to a combination of salinity and heat stress (Figs 3B and 7, S3 and S6–S8 Figs). Thus, little overlap was found between salt and heat combination-specific transcripts and drought and heat combination-specific transcripts (Fig 7), low representation was found for ethylene response transcripts in the salt and heat combination-specific transcripts (Fig 3B and S3 Fig), and knockout plants deficient in *mbf1c*, *apx1*, or *ain1* (involved in ethylene responses) did not show enhanced sensitivity to salt and heat combination (S6–S8 Figs). Together, these results suggest that significant differences exist in the acclimation response of Arabidopsis to different combinations of stresses (i.e. heat and drought versus heat and salinity). Meta-analysis of overlap between transcripts specific to different abiotic stress combination has indeed revealed very little overlap between the transcripts significantly upregulated in response to salt and heat stress combination (this study), and transcripts significantly up-regulated in response to other stress combinations (Fig 7; [7, 8, 52]). More studies are however needed to address the overlap between different abiotic stress combinations.

Our study reveals that different plant species could differ in their acclimation response to a combination of salinity and heat stress, highlights the unique role of ABA in the response of Arabidopsis plants to a combination of salt and heat stress, and demonstrates a high degree of specificity in the response of plants to different abiotic stress combinations.

### Accession Numbers

Arabidopsis Genome Initiative locus identifiers for genes mentioned in this article are as follows: ABA response protein (At3g02480), glyoxylase 17 (At1g80160), RbohD (At5g47910), NCED3 (At3g14440), Cat2 (At4g35090), Cor78 (At5g52310), APX1 (At1g07890) and MBF1c (At3g24500). RNA-Seq data from this study was deposited in NCBI GEO repository under the accession/reference number GSE72806.

## Supporting Information

S1 Fig Temperature and humidity measurements for the heat and heat and salinity combination treatments. (A) Temperature and humidity measurements over a 24 h period used in the 3 day stress treatment to monitor survival rate, growth parameters, and chlorophyll concentration. (B) Temperature and humidity measurements over a 4 h period used in the 1 h stress treatment to conduct RNA-Seq, qRT-PCR, and Na^+^ and K^+^ analyses. The temperature and humidity monitor was placed in and out of the chamber at the same time the plants were.
(TIF)

S2 Fig Expression of selected transcripts in response to salt, heat and a combination of salt and heat stress measured with qPCR. (TIF)

S3 Fig Proportion of ABA, GA and SA response transcripts in the data sets of transcripts specifically upregulated in response to salt, heat and a combination of salt and heat stress. (TIF)

S4 Fig Acclimation of *sid2* plants subjected to salt, heat and a combination of salt and heat stress. (TIF)

S5 Fig Acclimation of *lox3* plants subjected to salt, heat and a combination of salt and heat stress. (TIF)

S6 Fig Acclimation of *ain1-1* plants to salt, heat and a combination of salt and heat stress. (TIF)

S7 Fig Acclimation of *mbf1c* plants to salt, heat and a combination of salt and heat stress. (TIF)

S8 Fig Acclimation of *apx1* plants to salt, heat and a combination of salt and heat stress. (TIF)

S9 Fig Absolute values for Fig 5A. (TIF)

S10 Fig Absolute values for Fig 6A. (TIF)

S1 Table Transcripts significantly up-regulated in response to heat stress. (XLSX)

S2 Table Transcripts significantly up-regulated in response to salt stress. (XLSX)

S3 Table Transcripts significantly up-regulated in response to a combination of salt and heat stress. (XLSX)

S4 Table Transcripts significantly up-regulated in response to salt and heat stress but not salt and heat stress combination. (XLSX)

S5 Table Transcripts significantly up-regulated in response to salt and a combination of salt and heat stress. (XLSX)

S6 Table Transcripts significantly up-regulated in response to heat and a combination of salt and heat stress. (XLSX)

S7 Table Transcripts significantly up-regulated in response to salt, heat and a combination of salt and heat stress. (XLSX)

S8 Table Transcripts significantly down-regulated in response to heat stress. (XLSX)

S9 Table Transcripts significantly down-regulated in response to salt stress. (XLSX)

S10 Table Transcripts significantly down-regulated in response to a combination of salt and heat stress. (XLSX)

S11 Table Transcripts significantly down-regulated in response to salt and heat stress. (XLSX)

S12 Table Transcripts significantly down-regulated in response to salt and a combination of salt and heat stress. (XLSX)

S13 Table Transcripts significantly down-regulated in response to heat and a combination of salt and heat stress. (XLSX)

S14 Table Transcripts significantly down-regulated in response to salt, heat and a combination of salt and heat stress. (XLSX)

S15 Table Transcripts specifically up-regulated by a combination of salt and heat stress. (XLSX)

S16 Table Primer pairs for qRT-PCR. (XLSX)

## Abbreviations

ABA: abscisic acid
ACC: 1-Aminocyclopropane-1-carboxylic acid
APX1: ascorbate peroxidase 1
BL: brassinolide
CK: cytokinin
GA: gibberellin
IAA: indoleacetic acid
MBF1c: multiprotein bridging factor 1c
MJ: Methyl Jasmonate
SA: salicylic acid
