# Supplementary figures and images for: ABA Is Required for Plant Acclimation to a Combination of Salt and Heat Stress
Source: PLoS One. 2016 Jan 29;11(1):e0147625. doi: 10.1371/journal.pone.0147625 (PMC4733103; doi:10.1371/journal.pone.0147625)

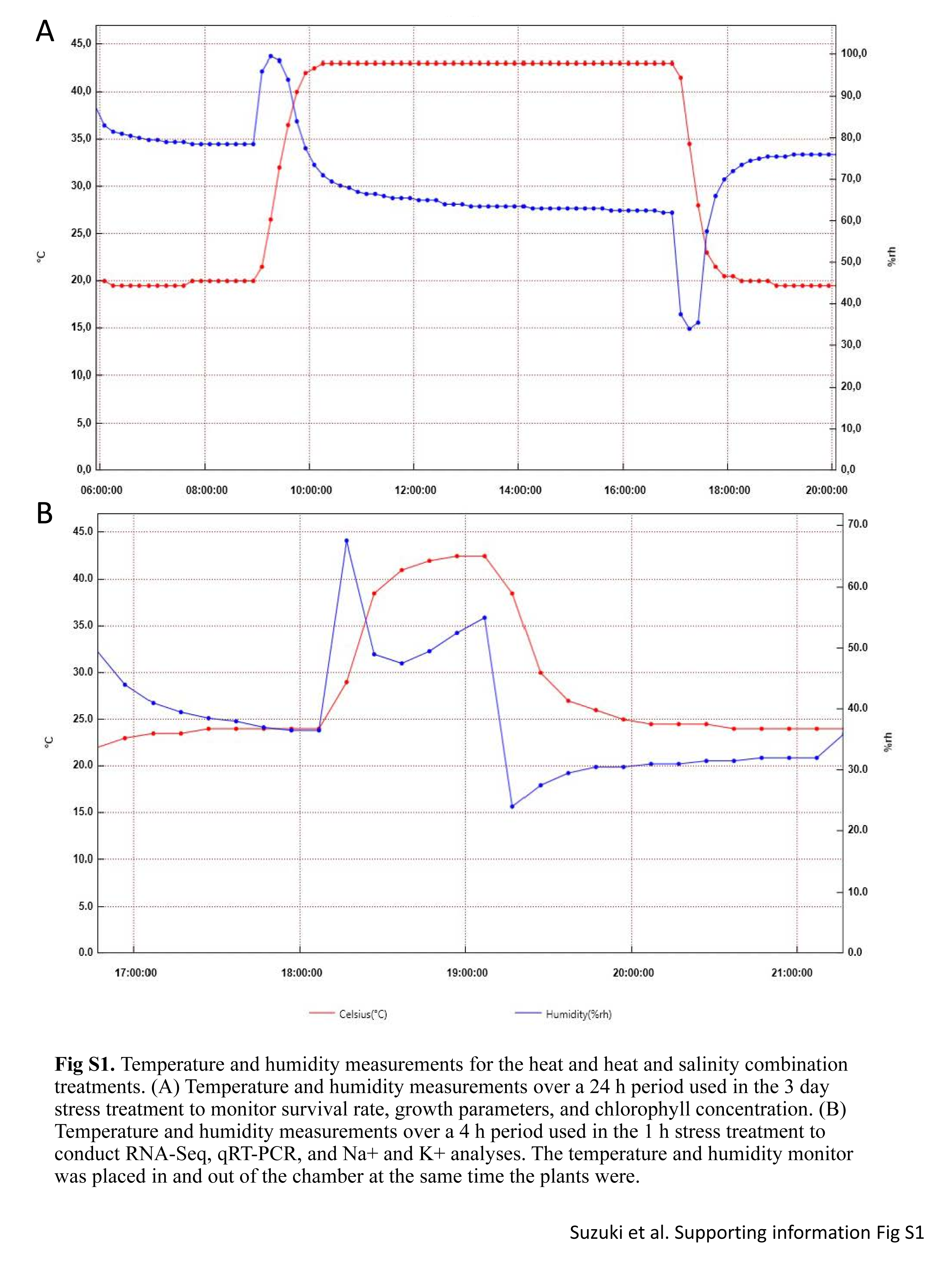

Supplement: S1 Fig — (A) Temperature and humidity measurements over a 24 h period used in the 3 day stress treatment to monitor survival rate, growth parameters, and chlorophyll concentration. (B) Temperature and humidity measurements over a 4 h period used in the 1 h stress treatment to conduct RNA-Seq, qRT-PCR, and Na+ and K+ analyses. The temperature and humidity monitor was placed in and out of the chamber at the same time the plants were. (TIF) [file pone.0147625.s001.tif]

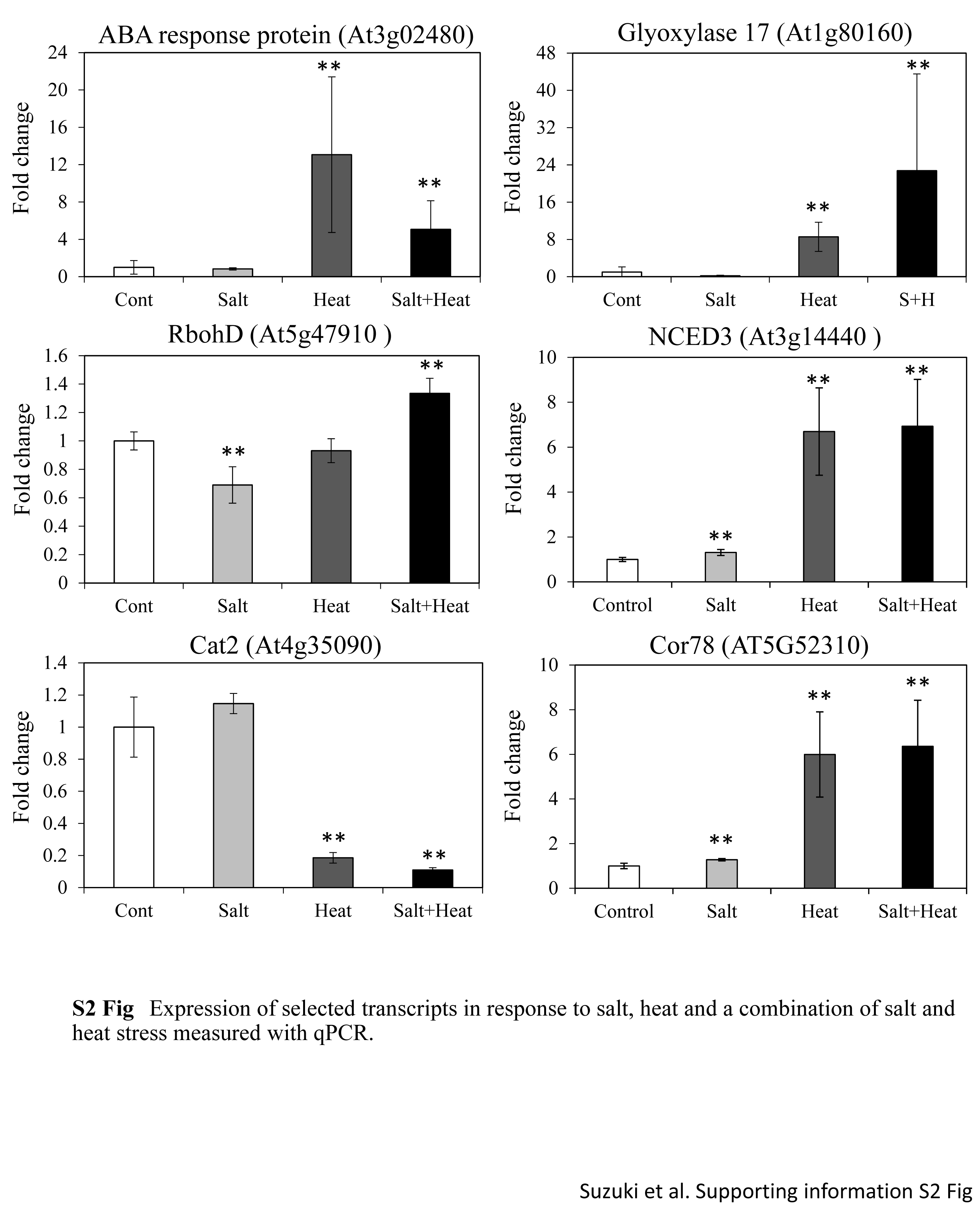

Supplement: S2 Fig — (TIF) [file pone.0147625.s002.tif]

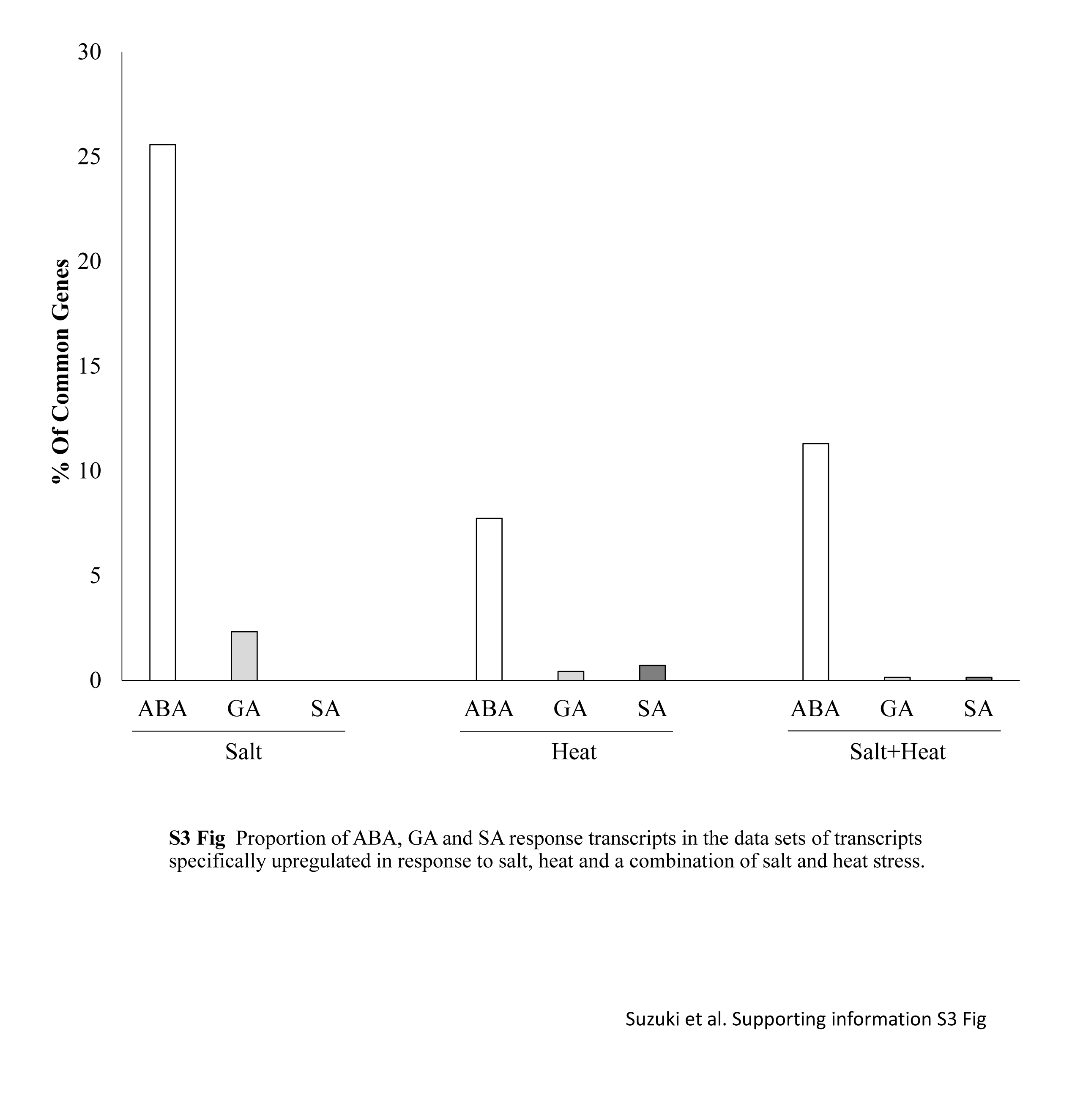

Supplement: S3 Fig — (TIF) [file pone.0147625.s003.tif]

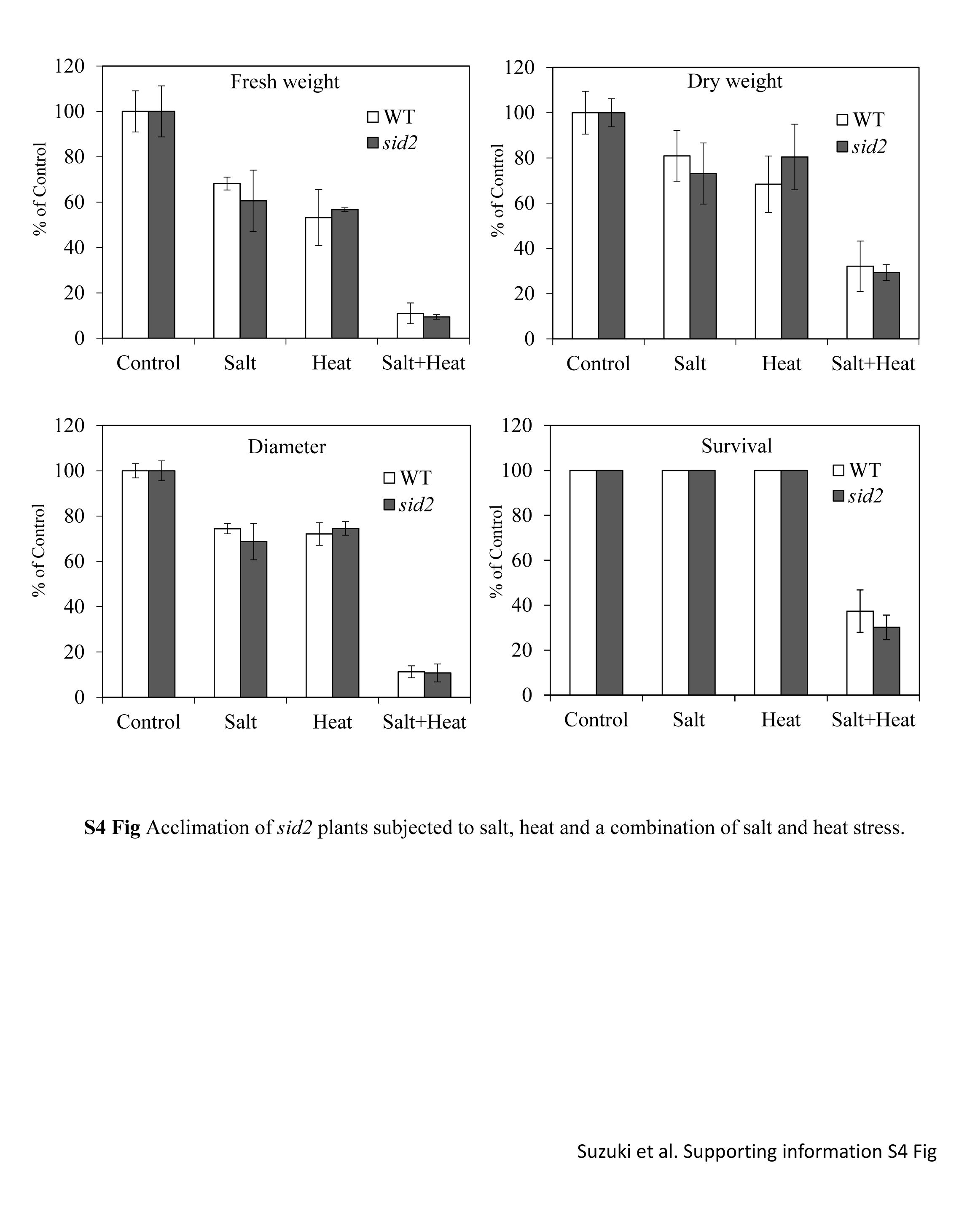

Supplement: S4 Fig — (TIF) [file pone.0147625.s004.tif]

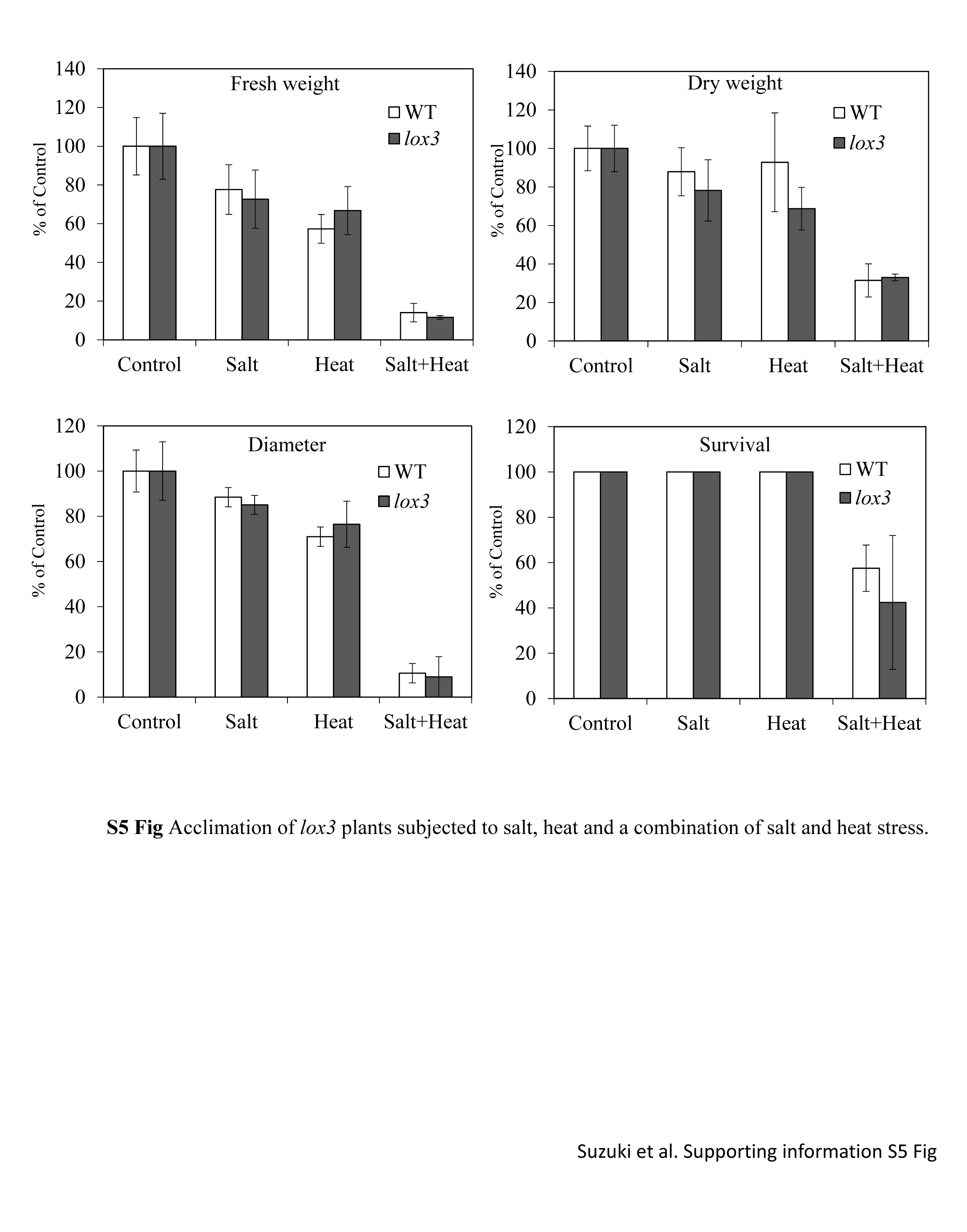

Supplement: S5 Fig — (TIF) [file pone.0147625.s005.tif]

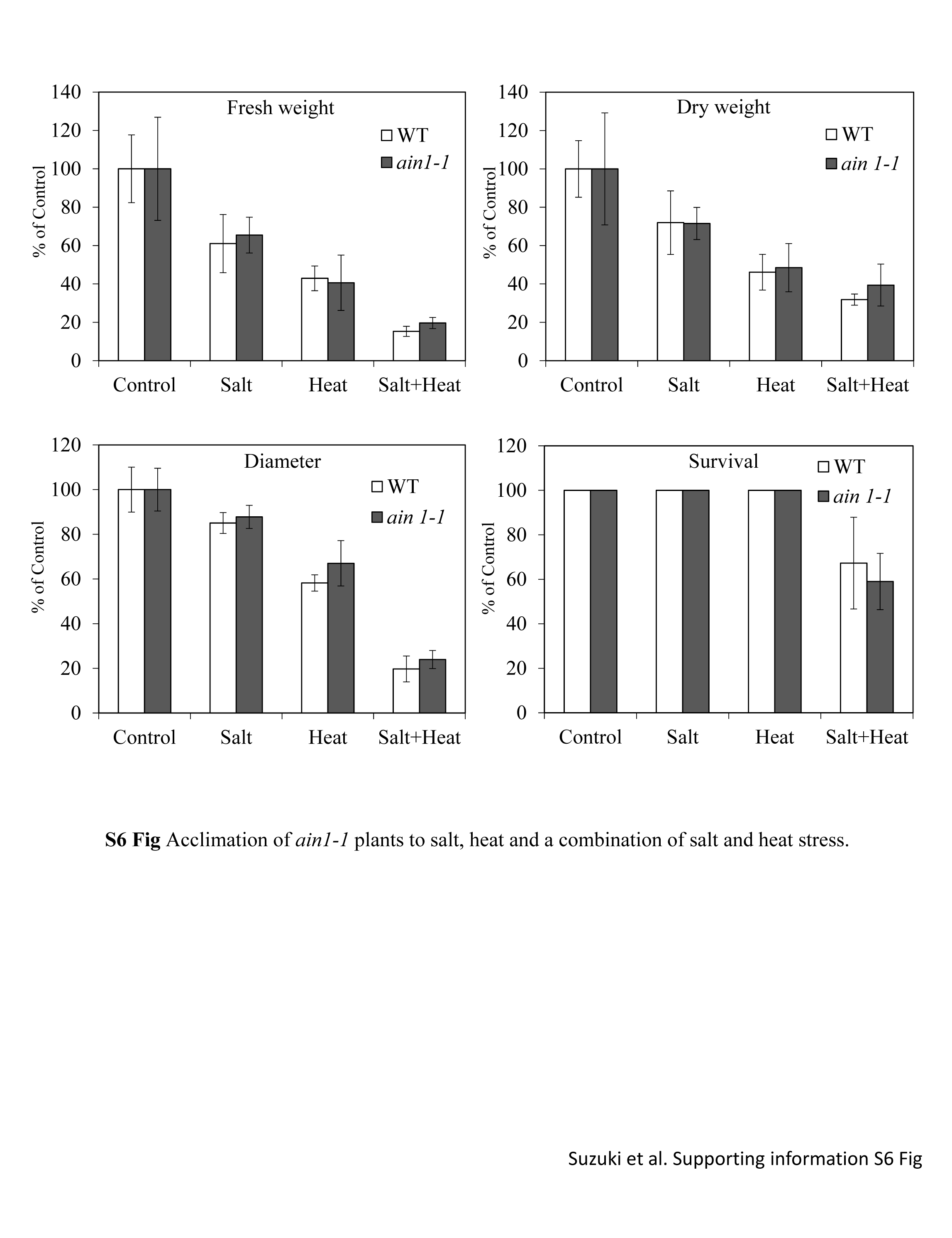

Supplement: S6 Fig — (TIF) [file pone.0147625.s006.tif]

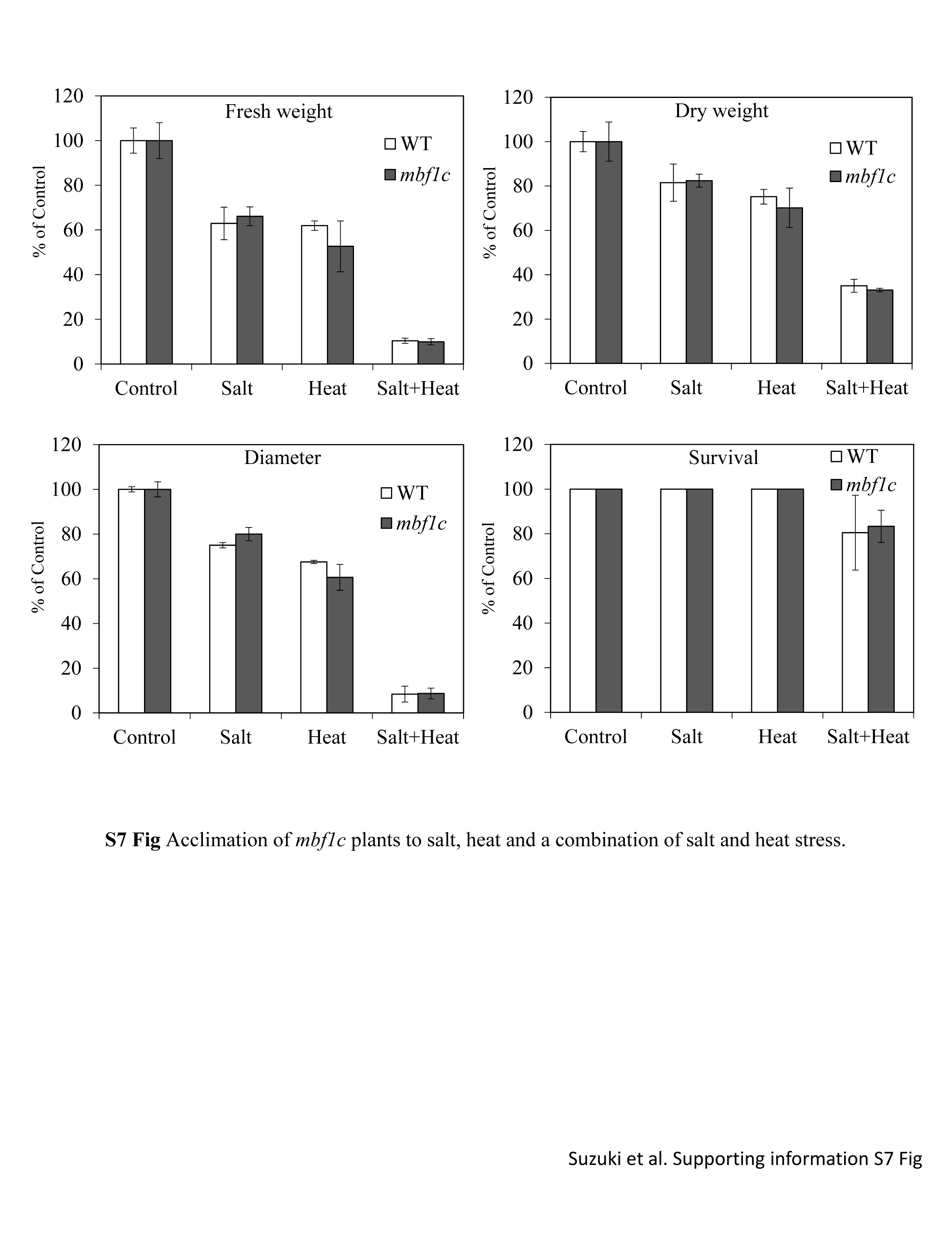

Supplement: S7 Fig — (TIF) [file pone.0147625.s007.tif]

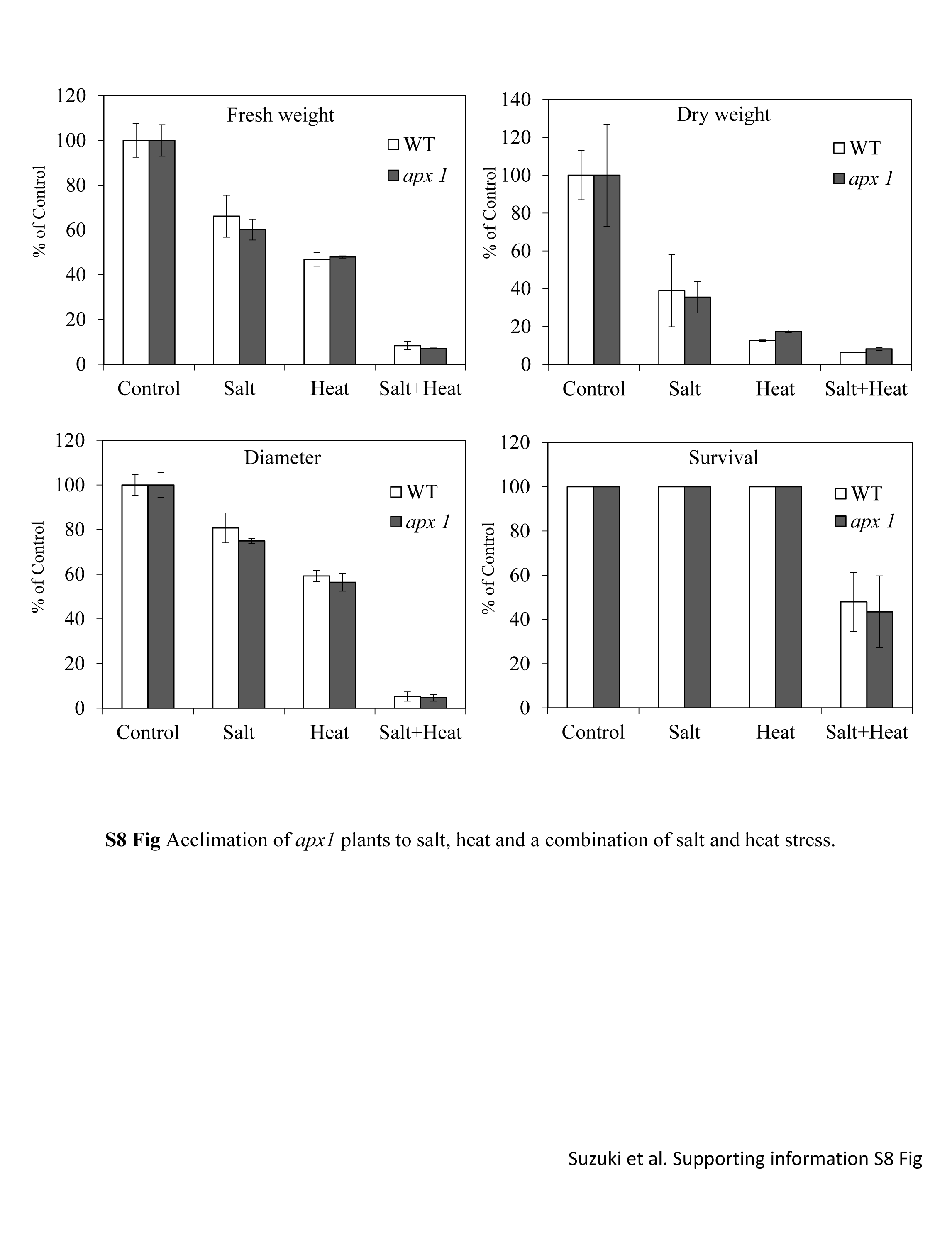

Supplement: S8 Fig — (TIF) [file pone.0147625.s008.tif]

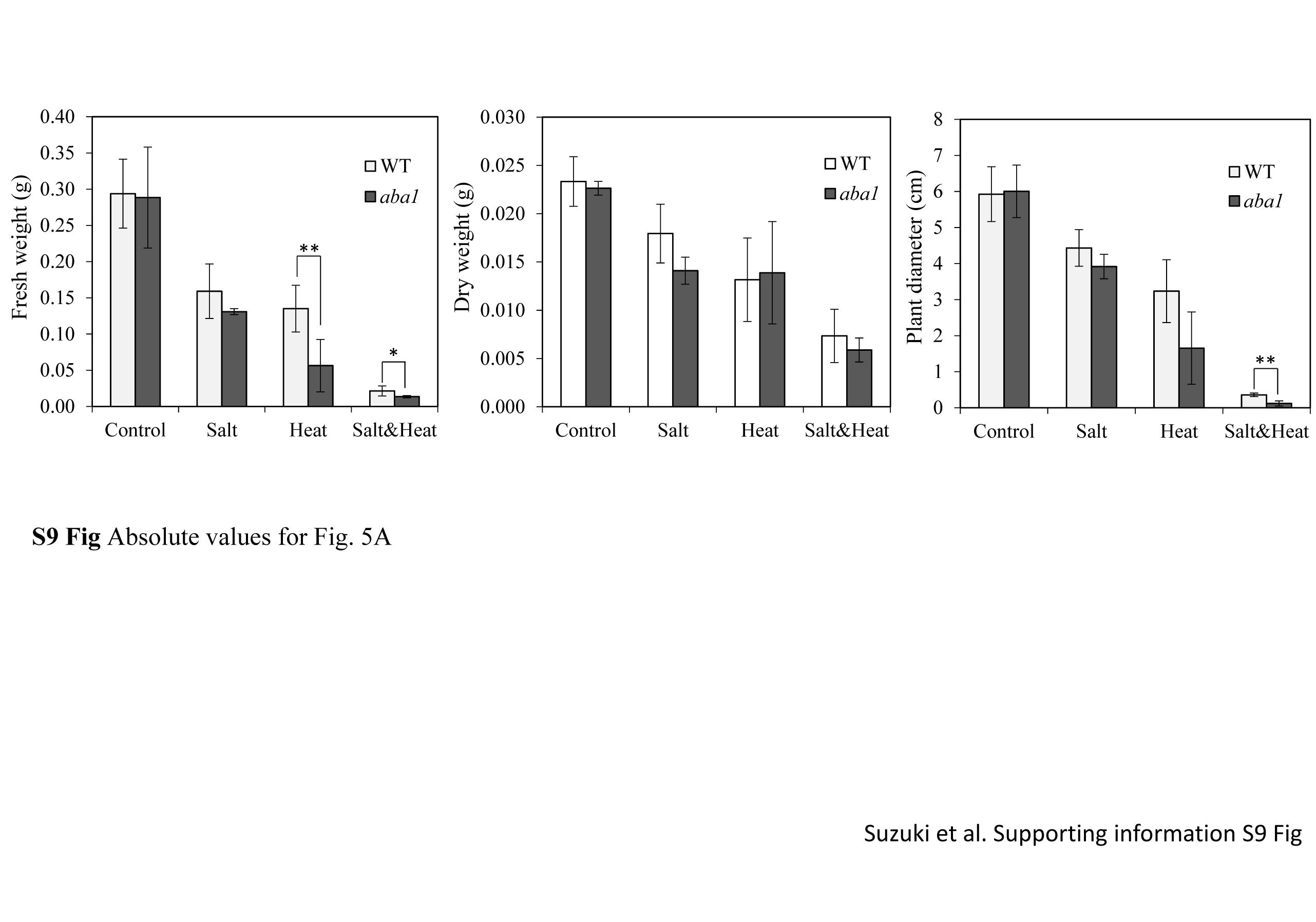

Supplement: S9 Fig — (TIF) [file pone.0147625.s009.tif]

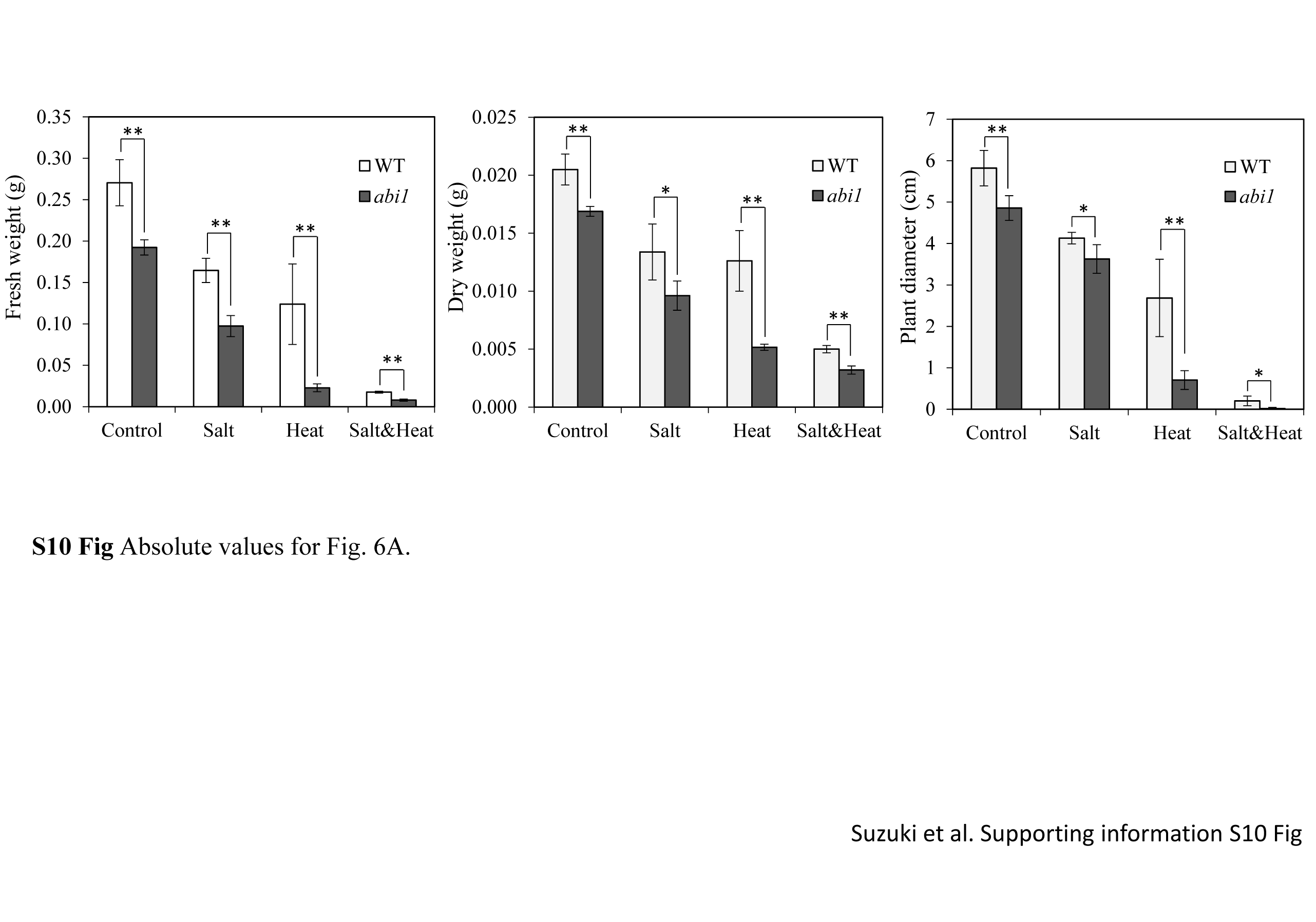

Supplement: S10 Fig — (TIF) [file pone.0147625.s010.tif]
